# A Comparative Transcriptomics Approach to Analyzing the Differences in Cold Resistance in *Pomacea canaliculata* between Guangdong and Hunan

**DOI:** 10.1155/2020/8025140

**Published:** 2020-08-03

**Authors:** Jing Liu, Zhiying Sun, Zhi Wang, Yuande Peng

**Affiliations:** ^1^College of Resources and Environment, Hunan Agriculture University, Changsha, 410128 Hunan, China; ^2^College of Bioscience and Biotechnology, Hunan Agriculture University, Changsha, 410128 Hunan, China; ^3^College of Life Sciences, Hunan Normal University, Changsha, 410081 Hunan, China; ^4^Institute of Bast Fiber Crops, Chinese Academy of Agricultural Sciences, Changsha, 410205 Hunan, China

## Abstract

*Pomacea canaliculata*, known as an invasive freshwater snail, is also called a golden apple snail; its survival and expansion are greatly affected by temperature. In this study, high-throughput sequencing (RNA-seq) was used to perform comparative transcriptome analysis on the muscular tissue (G_M) of snails in Guangdong and Hunan. Differential gene screening was performed with FDR <0.05 and |log2FoldChange| >1 as the threshold, and a total of 1,368 differential genes were obtained (671 genes showed upregulation in snails from Guangdong, and 697 genes displayed upregulation in snails from Hunan). Fifteen genes were identified as candidate genes for the cold hardiness of *Pomacea canaliculata*. Among them, three genes were involved in energy metabolism (glycogen synthase, 1; DGK, 1; G6PD, 1); seven genes were involved in homeostasis regulation (HSP70, 2; BIP, 1; GPX, 1; GSTO 1, G6PD, 1; caspase-9, 1); two genes were involved in amino acid metabolism (glutamine synthetase, 1; PDK, 1); and four genes were involved in membrane metabolism (inositol-3-phosphate synthase, 1; Na^+^/K^+^-ATPase, 1; calcium-binding protein, 2). This study presents the molecular mechanisms for the cold hardiness of *Pomacea canaliculata*, which could provide a scientific basis for the forecast and prevention of harm from *Pomacea canaliculata*.

## 1. Introduction

*Pomacea canaliculata* is native to freshwater wetlands in South America. In the early 1980s, it was introduced to China and other Asian countries from Argentina via Taiwan for commercial purposes. Due to its poor taste and poor market prospects, *P. canaliculata* gradually lost its commercial value and was primarily discarded and eventually colonized in the natural environment. *P. canaliculata* has caused considerable losses to the agricultural economy and threatens ecological security because of its characteristics of miscellaneous feeding, strong environmental adaptability, strong reproduction ability, and a lack of natural enemies. This makes *P. canaliculata* the only freshwater snail among the 100 worst invasive species in the world [1]. *P. canaliculata* has formed natural populations in most areas of southern China. With environmental adaptation and climate change, it will further expand north, which may cause more significant harm [2]. Previous research has shown that low temperature in winter is a significant factor in limiting the expansion of apple snails [3]. There have also been many studies on the hypothermic physiological response of mollusks. For example, taurine and strombine in *Mytilus edulis* may likely be cryoprotectants [4]. *Melampus bidentatus* can reduce its supercooling point by increasing the content of glycerol and proline in the organism [5]. The secretion of an epiphragm also allows mollusks to minimize the possibility of inoculative freezing and enhances cold tolerance [6]. In the wild, the cold hardiness of *P. canaliculata* increases with the decrease in temperature before winter approaches [7]; however, the molecular mechanism is unclear. Many properties of *P. canaliculata*, such as cold tolerance and immunological properties, are controlled by genes [8]. To elucidate the mechanism of regulation, various studies have been carried out.

According to an earlier study, the tolerance of mollusks is closely related to the metabolic performance of the organisms. The main goal of adaptations of the energy metabolism is to restore the metabolic balance to maximize stress tolerance [9]. The metabolic activity of carbohydrates may also be an essential physiological mechanism for cold hardiness for *P. canaliculata*. A low temperature reduces the fluidity of membrane-bound proteins, which changes the higher-order structure and activity of protein ions and disrupts the pathways of the intracellular metabolism [7]. However, metabolic disorders related to cellular energetics are one of the leading causes of indirect chilling injury in animals. Some scholars also believe that glycogen and lipids as energy storage materials provide energy for many animals to overwinter [5].

Different studies have been proposed to determine whether cellular environmental homeostasis is a necessary condition for maintaining normal physiological functions and even the organism's normal life activities. The homeostasis regulatory system of *P. canaliculata* includes the unfolded protein response (UPR), an antioxidant system, and an apoptotic pathway. Stimulated by excessive stress signals, unfolded proteins or misfolded proteins accumulate in the endoplasmic reticulum (ER), thereby impairing normal physiological functions [10]. Yao and Somero proved that low temperatures can induce *Mytilus* to activate ER stress through the p38 MAPK pathway [11]. The ER responds to the stress by activating intracellular signal transduction pathways [12]. Excessive production of reactive oxygen species (ROS) can cause cellular metabolic disorders or membrane damage [13–15]. Cold stress causes oxidative stress in the organism, mainly caused by the accumulation of ROS [16], while antioxidant enzymes in the organism can remove them. Apoptosis has emerged as a critical regulator in the physiological growth control and regulation of tissue homeostasis, which is the intrinsic death program of cells. It can also promptly remove excess and injured cells from the organism [17].

Some studies point out that *Helix Aspera*'s ability to supercool is related to water decrease and osmotic pressure increase in the body, and *P. canaliculata* may have the same correlation [5, 18]. Current research indicates that most freshwater invertebrates have developed the ability to regulate osmolality (where the osmolality of the hemolymph is above ambient water pressure), and *P. canaliculata* has also proved that it is moderately hypertonic [19]. Free amino acids help regulate the intracellular osmolality and cell volume of oysters [20], while the study of *Pomacea bridgesii* has illustrated that the content of some amino acids in the gills will increase with increasing osmolality [21].

The cell membrane is the first barrier for the cell and organelle in contact with outside, and its destruction is often an essential cause of the death of organisms at low temperatures [22]. Most organisms can increase their survival rate by increasing the unsaturated fatty acid content of membrane lipids at a low temperature, so that the membrane can maintain its normal physiological functions. Ca^2+^ can bind to the cell membrane and help resist the indirect damage caused by cell shrinkage or prevent the deformation of the compound in the membrane through its physical stability. This activity could reduce cell damage to mollusks during freezing [6].

Studies on the cold hardiness of *P. canaliculata* mainly focus on environmental factors and low-molecular-weight compounds, but few studies focus on molecular factors. So far, only HSP70, NKA, GK, and GPDH have been reported as related to the cold hardiness of *P. canaliculata* [23]. In the present study, *P. canaliculata* in Guangdong and Hunan were taken as research objects. The purpose was to identify the genes related to the cold hardiness of snails through transcriptome data and to explain the molecular relationship between cold hardiness and energy metabolism, cellular environmental homeostasis, amino acid metabolism, and cell membrane metabolism.

## 2. Materials and Methods

### 2.1. Experimental Materials

Samples of *P. canaliculata* used in this research were collected in December in the field. *P. canaliculata* in Guangdong were collected in the field ditch of Dongsheng Town, Zhongshan City, Guangdong Province (113°08′E, 28°17′N), and *P. canaliculata* in Hunan were collected in the pond of Hunan Agricultural University, Changsha City, Hunan Province (113°29′E, 22°61′N). The two breeds of snails were separately reared in aquariums (18 × 24 × 40 cm) with two liters of dechlorinated tap water. They were fed with cabbage and were kept at room temperature with a photoperiod of 16L : 8D. The experiment was approved by the Hunan Agricultural University Ethics Committee and followed the principles of the care and use of experimental animal management practices.

### 2.2. RNA Extraction and Transcriptome Sequencing

A total of six snails were selected as samples to do transcriptome sequencing. There are three active female snails from the Guangdong group and three other snails from the Hunan group. After dissecting the sampled snails, we quickly cut the muscle tissue of the foot from each snail. Then, we stored the cut samples at -80°C to freeze in liquid nitrogen, so we could extract RNA. The RNA easy Lipid Tissue Mini Kit (QIAGEN, Germany) was used to extract the total RNA from each sample and to establish a separate gene pool for each. A NanoPhotometer, spectrophotometer, Qubit2.0 Fluorometer, and Agilent 2100 bioanalyzer method were used to detect the purity, concentration, and integrity of each RNA sample. Preliminary quantification using the Qubit2.0 Fluorometer was performed after constructing the library. Then, the insert size of the library was detected using the Agilent 2100 bioanalyzer. To quantify the effective library concentration accurately to ensure library quality, we used qRT-PCR. The Illumina HiSeq2500 platform was used for high-throughput sequencing, with a sequencing read length of 150 bp.

### 2.3. Sequencing Data Processing and Statistical Analysis

Raw reads were sequenced to filter out reads with adapters, reads with N, and low-quality reads. Then, we could obtain clean reads. The comparison was conducted using the TopHat software after downloading the reference genome from the website (https://www.ncbi.nlm. http://nih.gov/genome/?term=Pomacea+canaliculata). DESeq2 R software (1.16.1) was used to screen the differential expression of genes in the study. The screening conditions were FDR <0.05 and |log2FoldChange| >1. Then, we drew a volcano map of the differentially expressed genes and performed the cluster analysis. Data were processed by Microsoft Excel software (SPSS V. 17.0 software was used for statistical analysis).

### 2.4. GO Function Annotation and KEGG Pathway Enrichment Analysis of Differential Genes

Using the Cytoscape plug-in ClueGo + Cluepedia, we aimed to do gene ontology (GO) classification of the differential genes, and we adopted the software default settings as the analysis parameter settings. Next, the result was displayed when *p* < 0.05, with a medium network specificity. The pathway analysis of the differential genes was completed using the Kyoto Encyclopedia of Genes and Genomes (KEGG) database (*p* < 0.05).

## 3. Results

### 3.1. Analysis of Sequencing Data

We obtained a total of 288,438,580 clean reads after processing the sequencing data. According to the statistics, the clean bases of each sample were above 6.43 Gb, and their Q30 was above 93.37%. The comparison ratio with the reference genome was between 80.83% and 83.77%, and the unique mapped ratio was higher than 79.09% (see Table 1), indicating that the sequencing results could be used for the subsequent analysis.

### 3.2. Screening and Cluster Analysis of Differentially Expressed Genes

**Figure 1:**
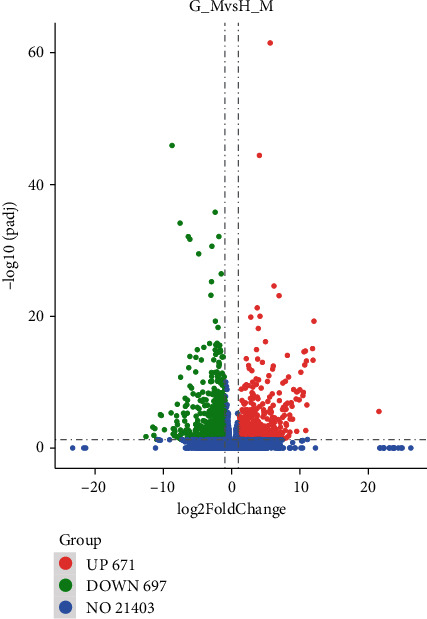
Volcano plot of differentially expressed genes.

**Figure 2:**
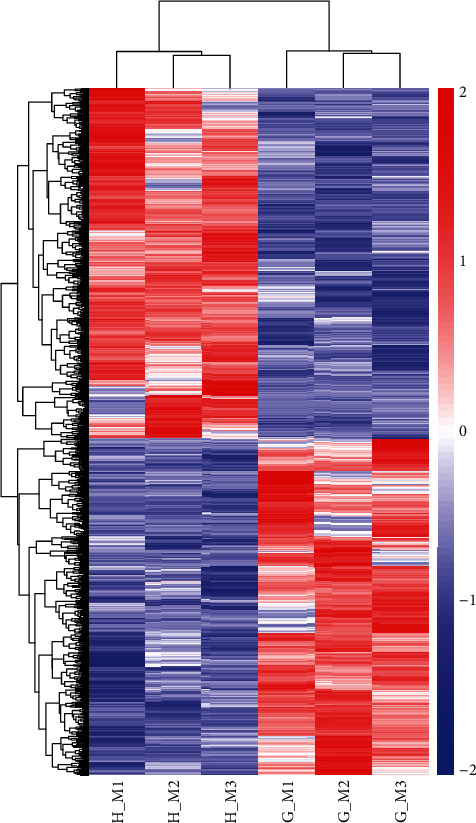
Heat map of differentially expressed genes.

Through differential expression analysis, the *P. canaliculata* in Guangdong and Hunan were compared, and a total of 1,368 differentially expressed genes were obtained. Among them, 697 differential genes showed downregulation in Guangdong snails, and 671 genes showed upregulation. The volcano map (Figure 1) shows a quick view of the distribution of the difference in expression levels between the two groups of samples. Cluster analysis of differentially expressed genes (Figure 2) found that the three biological duplicates of the snails in the same area gathered, indicating that the samples used in this study had excellent biological repeatability, and the sample grouping was much reasonable.

### 3.3. Analysis of Molecular Functions, Biological Processes, Cellular Components, and Pathway Enrichment of Differential Genes

**Figure 3:**
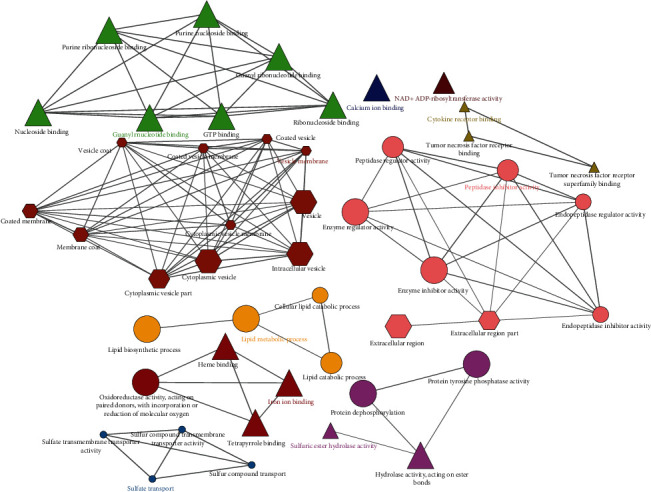
Network of the significantly enriched gene ontology (GO) term. Ellipses, triangles, and hexagons represent BP, MF, and CC, respectively.

The 47 GO terms were divided into ten groups and were connected by 78 edges. The essential terms included lipid metabolic process, sulfate transport, guanyl nucleotide binding, iron ion binding, vesicle membrane, cytokine receptor binding, sulfuric ester hydrolase activity, peptidase inhibitor activity, calcium ion binding, and NAD^+^ ADP-ribosyltransferase activity (Figure 3). Terms based on the kappa scoring level (≥0.1) served as a functional grouping network of connected nodes. For each group, the size of the nodes indicates their importance, with the most important path having the largest node size.

**Figure 4:**
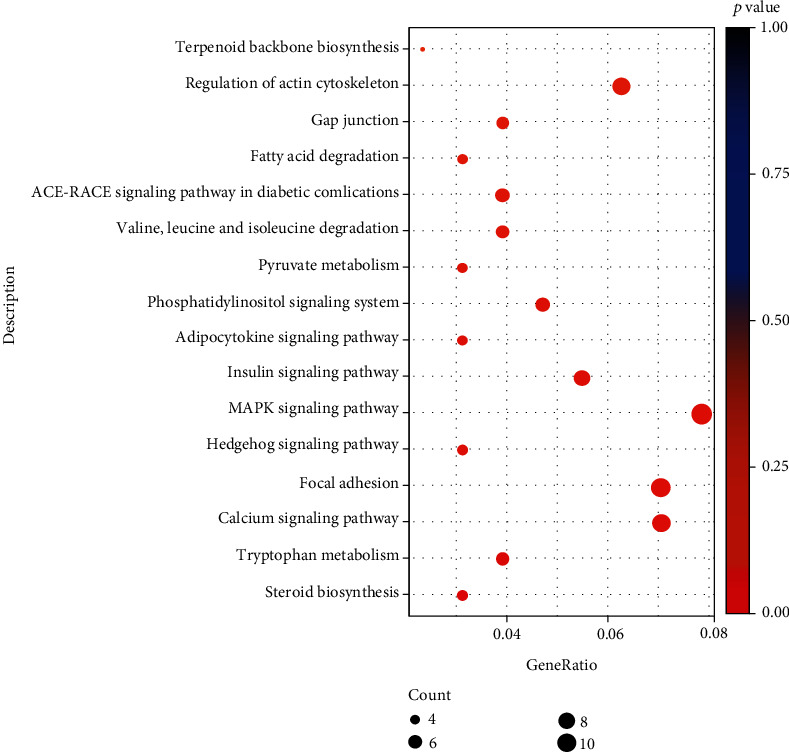
The significantly enriched Kyoto Encyclopedia of Genes and Genomes (KEGG) term of differentially expressed genes.

Differential genes were annotated to 112 pathways of KEGG, of which 16 were significantly enriched pathways (*p* < 0.05), for example, steroid biosynthesis, tryptophan metabolism, and calcium signaling pathway (Figure 4).

### 3.4. Identification of Candidate Cold-Resistance Genes

**Figure 5:**
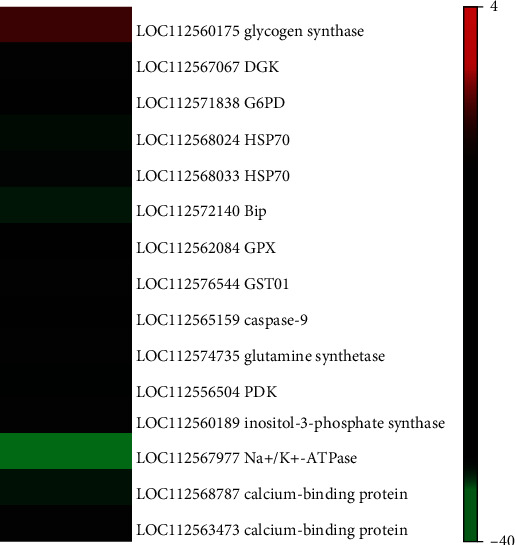
Expression profile of candidate genes for cold resistance in *Pomacea canaliculata.*

We identified a total of fifteen genes as the candidate cold-resistance genes of *P. canaliculata* (Figure 5). These genes were related to energy metabolism, homeostasis regulation, amino acid metabolism, and cell membrane metabolism.

According to the test results, the glycogen synthase gene in Guangdong snails upregulated two times more than did the same genes in Hunan snails, but the DGK and G6PD genes downregulated three times less and two times less than did the same genes in Hunan snails. These three genes are involved in energy metabolism. Among the genes of homeostasis regulation, the first and second HSP70 genes in Guangdong snails downregulated eleven times and six times more than that in Hunan snails, respectively; the BIP gene downregulated eighteen times more than the BIP in Hunan snails; and the GPX, GSTO1, G6PD, and caspase-9 genes all downregulated two times more than did the same genes in Hunan snails. With regard to the genes involved in amino acid metabolism, the downregulation of glutamine synthetase gene and PDK gene in Guangdong snails were four times and two times more than that in Hunan snails. An inositol-3-phosphate synthase gene, a Na^+^/K^+^-ATPase gene, and two calcium-binding protein genes are related to membrane metabolism, and they all displayed downregulation in Guangdong snails compared to the same gene in Hunan snails. The downregulation of inositol-3-phosphate synthase gene was three times; the downregulation of Na^+^/K^+^-ATPase gene was more than thirty-eight times; the downregulation of the first and second calcium-binding protein genes were more than fifteen times and three times, respectively.

## 4. Discussion

*P. canaliculata* was introduced from Guangdong to Hunan in the mid-1980s, so the two breeds of snails have a high degree of homology and have a comparative basis. The weather in Guangdong Province is humid and warm throughout the year, with an average annual temperature of 19–24°C and an average temperature of 13.4°C in January. The daily average temperature is rarely below 0°C. However, the average annual temperature in Hunan Province is 16–19°C, and the average temperature in January is 4°C. The daily average temperature is usually below 0°C. Generally speaking, populations in cold regions at high latitudes have a stronger ability to withstand low temperatures. This differentiation is the result of the long-term adaptation of *P. canaliculata* to the invaded land under natural selection, and this adaptation may be related to genetic differentiation.

### 4.1. Candidate Cold-Resistance Genes Related to Energy Metabolism

Some earlier studies have shown that carbohydrates and lipid metabolism could provide energy for snails to overwinter. Glycogen, known as an energy storage substance, is also gradually reduced in the organism, as a result of its body energy consumption [5]. Glycogen synthase is a critical enzyme in glycogenesis during glycogen accumulation [24]. At low temperatures in winter, the expression of the glycogen synthase gene in *Crassostrea gigas* has a negative correlation with glycogen content [25]. In our study, a glycogen synthase gene was observed to be upregulated in the Guangdong snails. Diacylglycerol kinase delta (dgk*δ*) could significantly contribute to adipogenesis, and a downregulated DGK gene also exists in the snails from Guangdong. The pentose phosphate pathway (PPP), which commonly exists in animals, plants, and microorganisms, is a sugar metabolic pathway that can generate energy [26]. The enzyme encoded by the glucose 6-phosphate dehydrogenase (G6PD) gene is the first step responsible for catalyzing the PPP [27, 28]. The present study also found a gene, G6PD, which showed downregulation in the snails from Guangdong.

Based on these findings, we believe that Guangdong snails possibly reduce the accumulation of energy-supplying substances and cold resistance by upregulating the glycogen synthase gene and downregulating the DGK gene; downregulating the G6PD gene is helpful in reducing energy metabolism efficiency, thereby reducing the cold resistance of the snail. Therefore, glycogen synthase, DGK, and G6PD genes are the critical energy metabolism genes that control the cold resistance of *P. canaliculata*.

### 4.2. Candidate Cold-Resistance Genes Related to Homeostasis Regulation

That the maintenance of intracellular homeostasis improves the cold resistance of snails has been proved in a previous study. HSP70, as a molecular chaperone in cellular protein folding, stabilizes the natural conformation of the protein and promotes the correct folding of protein subunits, and it is closely related to the antistress ability of the organism [9]. A study also indicates that the transcript-level of HSP70 is promoted along with the low-temperature stimulation [23]. In this paper, two HSP70 genes showed downregulation in snails from Guangdong. BIP is a member of the ER heat shock protein family. As a chaperone protein, it plays an essential role in helping proteins fold correctly and in the process of degrading the wrong proteins [29, 30]. Some reports have pointed out that the upregulation of the BIP chaperone protein is part of the adaptive cellular response to ER stress [31]. In our experiment, one BIP gene was downregulated in Guangdong snails.

Glutathione peroxidase (GPX) is an antioxidant enzyme that scavenges reactive oxygen species, and *C. gigas* enhances antioxidant capacity by upregulating the GPX [32, 33]. In this study, one GPX gene was observed with downregulation in Guangdong snails. Glutathione S-transferase omega-1 (GSTO 1) has the effect of scavenging reactive oxygen species and protecting the organism from oxidative stress [34]. *Mytilus edulis* increases antioxidant capacity by upregulating the GSTO1, and in our study, one GSTO1 gene showed downregulation in the Guangdong snail. The NADPH produced by the PPP reduces when the glucose 6-phosphate dehydrogenase (G6PD) is insufficient and the content of reduced glutathione is lower than normal, which leads to the capacity of antioxidants to reduce [35]. In our experimental results, one G6PD gene in snails from Guangdong showed downregulation.

Caspase-9 is necessary for the process of apoptosis [36, 37]; the caspase protein shows a high expression level in the *mytilus galloprovincialis*, which makes the process of apoptosis very active [38]. One caspase-9 gene in snails from Guangdong was downregulated in our study.

For the above reasons, we conclude the following. First, downregulating the HSP70 and BIP genes could reduce the response to UPR. Second, the antioxidant capacity of snails could be reduced by downregulating the GPX, GSTO 1, and G6PD genes. Third, the downregulation of the caspase-9 gene could reduce the apoptosis ability of snails. This means that a decrease in the ability to regulate the cellular environmental homeostasis leads to the decline of the cold hardiness characteristic. Therefore, the HSP70, BIP, GPX, GSTO 1, G6PD, and caspase-9 genes are the key homeostasis regulatory genes that control the cold resistance of *P. canaliculata*.

### 4.3. Candidate Cold-Resistance Genes Related to Amino Acid Metabolism

Previous studies suggested that *P. canaliculata* could increase the content of specific amino acids in the organism by promoting the hemolymph osmotic pressure and thereby improving its cold resistance. Pyruvate dehydrogenase kinase (PDK) could deactivate the pyruvate dehydrogenase (PDH) by catalyzing its phosphorylation [39], while PDH catalyzes the pyruvate so it is oxidative [40]. This means that the activity of PDK is positively correlated with pyruvate content. Pyruvate might also produce alanine through transamination. Studies have also found that when a *Pomacea bridgesii* is exposed to an environment with high osmotic pressure, the alanine content in the gills will increase [21]. In this paper, a PDK gene was found with downregulation in Guangdong snails. Glutamine synthetase may promote the synthesis of glutamine, and the snails might enhance their ability to resist cold by increasing the glutamine content in their bodies to overwinter [5]. In this study, a glutamine synthetase gene showed downregulation in Guangdong snails.

We found that downregulating the PDK and glutamine synthetase genes reduced the osmotic pressure of apple snails, which caused its cold hardiness to decrease. Therefore, PDK and glutamine synthetase genes were identified as the regulatory genes that control the amino acid metabolism with respect to cold resistance.

### 4.4. Candidate Cold-Resistance Genes Related to Membrane Metabolism

A study demonstrated that increasing the content of Ca^2+^ and unsaturated fatty acids in the cell membrane could improve the cold resistance of the snails. Inositol-3-phospholipid synthetase is the first step to catalyzing the inositol synthesis (inositol is found mainly in animal cells in the form of phospholipids), and unsaturated fatty acids are also an important part of cell membrane phospholipids. In the study, an inositol-3-phosphate synthase gene was found with downregulation. Some literature has noted that the Na^+^/K^+^-ATPase gene in the snail might play a critical role in response to low-temperature treatment [23]. The activity of the Na^+^/K^+^-ATPase gene in the gills and hepatopancreas of *Mytilus edulis* has a good correlation with phospholipid content [41]. In this research, a Na^+^/K^+^-ATPase gene was observed with downregulation in Guangdong snails.

We already know that upregulating the calcium-binding protein can improve the organism's absorption of Ca^2+^. In our study, two calcium-binding protein genes were downregulated in the Guangdong snails.

Therefore, downregulating the inositol-3-phosphate synthase and Na^+^/K^+^-ATPase genes could reduce the unsaturated fatty acid content in the cell membrane of the organism. Then, downregulating the calcium-binding protein gene would reduce the ability of the snails to absorb Ca^2+^, which would mean that the Ca^2+^ bound to the cell membrane would decline accordingly. Decreasing the content of unsaturated fatty acids and Ca^2+^ in the cell membrane would also reduce the stability of the snails' cell membrane, which might lead to a decline in cold resistance. Therefore, inositol-3-phosphate synthase, Na^+^/K^+^-ATPase, and calcium-binding protein genes are the principal regulating genes of membrane metabolism that control cold resistance.

## 5. Conclusion

Energy supply, tolerance ability, and supercooling are assumed to be the factors affecting the cold resistance of snails in this study. Therefore, cold resistance could be enhanced by increasing the energy supply, by tolerance ability, and by supercooling.

The supply of energy involves two main aspects: the synthesis of energy supply materials and the efficiency of energy supply material metabolism. In this study, there were three genes involved in energy supply (Glycogen synthase, 1; DGK, 1; G6PD, 1). Homeostasis regulation and membrane metabolism are the two aspects that affect the tolerance ability of *P. canaliculate*. The regulation of homeostasis could affect the magnitude of the ecological tolerance, and the metabolism of the cell membrane might affect the physical stability of the membrane. In this paper, eleven genes were involved in the tolerance of snails (HSP70, 2; Bip, 1; GPX, 1; GSTO 1, G6PD, 1; caspase-9, 1; inositol-3-phosphate synthase, 1; Na+/K+-ATPase, 1; calcium-binding protein, 2). Amino acid metabolism can affect osmotic pressure and then regulate supercooling. In this study, two genes were identified that were involved in the regulation of supercooling (glutamine synthetase, 1; PDK, 1). It should be noted that the G6PD gene could affect both the energy metabolism efficiency and the tolerance ability of *P. canaliculata*.

**Figure 6:**
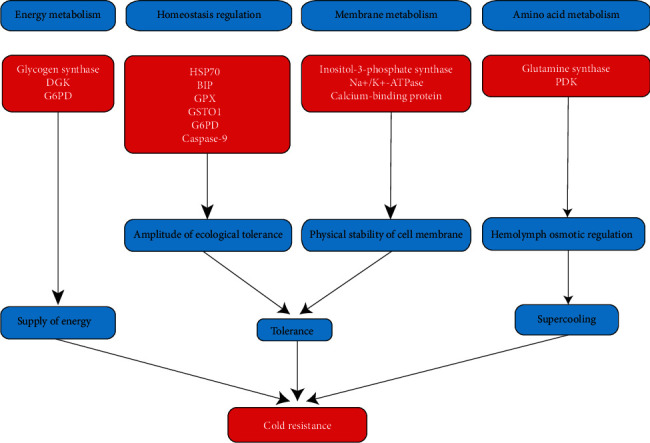
Hypothesized mechanisms of cold resistance in *Pomacea canaliculata.*

Based on the results of this research, we have established a hypothetical model describing the cold-resistance mechanism of *P. canaliculata* (Figure 6). The cold resistance of the snail is the main factor that affects its population distribution and the extent of the damage it does. Studying these snails' ability to resist cold provides a scientific basis for disaster warning and prevention. At the same time, the snails have characteristics such as fast reproduction, easy breeding, and strong tolerance. Therefore, *P. canaliculata* is expected to become a new model organism for studying mollusks. The results of the study on the cold resistance of *P. canaliculata* could provide a reference for other mollusk research.

## Figures and Tables

**Table 1:** Comparison results of clean reads and reference genome.

Sample number	Total reads	Mapped reads	Mapped ratio/%	Unique mapped reads	Unique mapped ratio/%
G_M1	46,181,938	38,474,909	83.31	37,631,049	81.48
G_M2	57,670,902	47,980,494	83.2	46,964,487	81.44
G_M3	45,769,776	38,341,279	83.77	37,554,261	82.05
H_M1	42,837,980	35,786,479	83.54	34,991,365	81.68
H_M2	48,782,384	39,761,112	81.51	38,996,115	79.94
H_M3	47,195,600	38,147,687	80.83	37,326,935	79.09

## Data Availability

The data of this study are available from the corresponding author.
